# Development of Poly(ɛ-Caprolactone) Scaffold Loaded with Simvastatin and Beta-Cyclodextrin Modified Hydroxyapatite Inclusion Complex for Bone Tissue Engineering

**DOI:** 10.3390/polym8020049

**Published:** 2016-02-09

**Authors:** Jung Bok Lee, Ji Eun Kim, Min Soo Bae, Su A Park, Daniel A. Balikov, Hak-joon Sung, Hoon Bong Jeon, Hun Kuk Park, Soong Ho Um, Kook Sun Lee, Il Keun Kwon

**Affiliations:** 1 Department of Biomedical Engineering, Vanderbilt University, Nashville, TN 37212, USA; jung.bok.lee@vanderbilt.edu (J.B.L.); daniel.a.balikov@vanderbilt.edu (D.A.B.); hak-joon.sung@vanderbilt.edu (H.S.); 2 Department of Mechanical Engineering, Vanderbilt University, Nashville, TN 37235, USA; 3 Department of Maxillofacial Biomedical Engineering, Institute of Oral Biology, School of Dentistry, Kyung Hee University, Seoul 130-701, Korea; sausage14@khu.ac.kr (J.E.K.); bmsbms20@gmail.com (M.S.B.); 4 Department of Nature-Inspired Nanoconvergence Systems, Korea Institute of Machinery and Materials, 156 Gajeongbuk-ro, Yuseong-gu, Daejeon 304-343, Korea; psa@kimm.re.kr; 5 Division of Cardiovascular Medicine, Vanderbilt University, Nashville, TN 37235, USA; 6 Department of Biomedical Engineering, College of Medicine, Kyung Hee University, 26, Kyungheedae-ro, Dongdaemun-gu, Seoul 130-701, Korea; inwoodkr@gmail.com (H.B.J.); sigmoidus@khu.ac.kr (H.K.P.); 7 School of Chemical Engineering & SKKU Advanced Institute of Nanotechnology, Sungkyunkwan University, Suwon, Gyeonggi-do 440-746, Korea; Sh.um@skku.ac.kr; 8 Department of Oral and Maxillofacial Radiology, College of Medicine, School of Dentistry, Kyung Hee University, Seoul 130-701, Korea; isodent@naver.com; 9 Severance Biomedical Science Institute, College of Medicine, Yonsei University, Seoul 120-752, Korea

**Keywords:** β-cyclodextrin, hydroxyapatite, poly(ɛ-caprolactone) 3-D scaffolds, simvastatin, bone regeneration

## Abstract

In this study, we developed poly(ɛ-caprolactone) (PCL) 3D scaffolds using a solid free form fabrication (SFF) technique. β-cyclodextrin (βCD) was grafted to hydroxyapatite (HAp) and this βCD grafted HAp was coated onto the PCL scaffold surface, followed by drug loading through an inclusion complex interaction between the βCD and adamantane (AD) or between βCD and simvastatin (SIM). The scaffold structure was characterized by scanning electron microscopy (SEM). The release profile of simvastatin in the β-CD grafted HAp was also evaluated. Osteogenic differentiation of adipose-derived stromal cells (ADSCs) was examined using an alkaline phosphatase activity (ALP) assay. The results suggest that drug loaded PCL-HAp 3-D scaffolds enhances osteogenic differentiation of ADSCs.

## 1. Introduction

In the field of tissue engineering, three-dimensional (3D) porous scaffolds have been fabricated to mimic extra cellular matrix (ECM) found in native tissue for regeneration, restoration, and repair of damaged tissue and organs [1,2,3]. As their ECM mimetic structures are considered to be critical for successful clinical applications, several rapid prototyping (RP) techniques have been developed including selective laser sintering (SLS) [4], stereolithography (SLA) [5], 3D printing [6] and solid free-form fabrication (SFF) [7,8,9]. In particular, the SFF technique can produce well-controlled, reproducible, scaffold structures such as pore size, porosity, and mechanical properties while varying their internal architecture [9,10,11].

Synthetic biodegradable polyesters such as poly(lactic-*co*-glycolic acid) (PLGA), poly(l-lactic acid) (PLLA), and poly(ɛ-caprolactone) (PCL) have been used as biomaterials in biomedical application due to their excellent biocompatibility and good mechanical properties. Among them, PCL is a thermosensitive polymer with low melting point of about 60 °C and a glass transition temperature of about −60 °C, a highly desirable set of temperatures. Its low melting point (60 °C) facilitates the SFF process compared to other polyesters. Furthermore, Several PCL-derived products have also been approved by the Food and Drug Administration (FDA) for clinical applications [12,13]. Although continuous progress has been made so far, PCL has not been applied extensively for bone regeneration, a niche field of tissue engineering that could benefit greatly from utilizing PCL [9,14].

In this study, hydroxyapatite (HAp) was used because it is an attractive material for bone tissue engineering due to its inorganic components boosting affinity for ECM proteins and induction of osteogenic differentiation and mineralization [15,16,17]. Since β-cyclodextrin (βCD) has been previously used for solubilizing and stabilizing drugs by host-guest type inclusion complexation [18], an inclusion complex system between βCD and adamantane (AD) was used to attach HAp onto the surface of the PCL scaffold. Inclusion complex systems have been widely used as a drug loading strategy that forms a bond between ferrocene and cyclodextrin [19]. In solution or in solid state, the cavity of the cyclodextrin generates a hydrophobic environment, thereby changing its physical and chemical interactions with a guest molecule (e.g., drug) [20,21]. For our study, the “driving force” of the complex formation is the fundamental interaction between the βCD as the host molecule and AD as the guest molecule. Using this inclusion complex, we produced an HAp-coated PCL scaffold as well as simvastin (SIM)-loaded on HAp that has been known to promote osteogenic differentiation of mesenchymal stem cell via Ras/Smad/Erk signaling derived from bone morphogenic protein (BMP)-2 treatment [22,23]. We used these scaffold templates to examine their osteoconductive/osteoinductive abilities in *in vitro* human adipose-derived stromal cell (hADSC) and *in vivo* animal models.

## 2. Experimental Section

### 2.1. Materials

Poly(ɛ-caprolactone), hydroxyapatite (HAp) nanoparticles (NPs), 3-aminopropyltriethoxysilane (APTES), ascorbic acid, dexamethasone, β-glycerophosphate (glycerol 2-phosphate disodium salt hydrate), and adamantylamine were purchased from Sigma-Aldrich (St. Louis, MO, USA). β-cyclodextrin (β-CD) was purchased from TCI (TOKYO Chemical Industry Co. Ltd., Tokyo, Japan). Dulbecco’s modified Eagle’s medium (DMEM), fetal bovine serum (FBS), phosphate buffered saline (PBS), trypsin-EDTA and antibiotics (penicillin-streptomycin) were purchased from Gibco (Rockville, MD, USA). Simvastatin was purchase from Wako (Osaka, Japan). StemPro^®^ Human Adipose-Derived Stem Cells (hADSCs) were obtained from Invitrogen (Carlsbad, CA, USA).

### 2.2. Poly(ɛ-caprolactone) (PCL) Scaffold Preparation

PCL scaffolds were created using a 3D SFF plotting system consisting of a 3-axes machine with a 10 × 10 × 10 cm *x*–*y*–*z* stage, dispenser, nozzle, compression/heat controller, and software/hardware system. PCL pellets were melted in a cylinder at 100 °C using the heating jacket of the plotting system. The hardware system controlled the scaffold structure by adjusting the pressure, feed rate, and nozzle size. The temperature, pressure, and feed rate were set at 85 °C, 650 kPa, and 150 µm/s, respectively. For the reaction with adamantine, acrylic acid (AAc) was grafted onto the surface of the PCL scaffold. Briefly, the scaffolds were wetted with 70% ethanol and then immersed in an aqueous AAc solution (10% in distilled water (DW) with 0.01 M ammonium ferrous sulfate) and exposed to gamma ray radiation (10 kGy) from a cobalt-60 source at ambient temperature. Scaffolds were washed with DW three times to remove any unreacted residual monomers or homoplymers. AAc-PCL scaffolds were immersed in adamantylamine (AD, 10 mM)/ethanol solution for 24 h with stirring to graft AD onto the PCL scaffold using 1-Ethyl-3-(3-dimethylaminopropyl)-carbodiimide (EDC)/N-hydroxysuccinimide (NHS). Finally, scaffolds were washed with DW three times and freeze dried.

### 2.3. Preparation of β-Cyclodextrin (βCD) Grafted Hydroxyapatite (HAp)

βCD (10 mmol) was dissolved in anhydrous toluene. Then 15 mmol succinic anhydride was added to the solution and the polymers were reacted with stirring at 60 °C for 12 h under N_2_. After the reaction, the product was concentrated by rotary-evaporation. The polymer was then precipitated in diethyl ether. For amino-functionalization of HAp, 5 g of HAp NPs were immersed in 100 mL anhydrous toluene containing 10 mL APTES at 120 °C under an N_2_ atmosphere with a reflux condenser for 24 h. The samples were then rinsed for 1 h in toluene to remove any unreacted silane, and βCD was grafted onto the surface of the HAp by a 1-ethyl-3-dimethylaminopropyl carbodiimide- (EDC) mediated reaction between the primary amine groups of the HAp NP surface and the carboxyl group of βCD. In order to quantify the amine concentration in the HAp surface, the HAp NPs were immersed in 500 µM acid orange 7 solution at pH 3 for 3 h at room temperature and then washed three times with pH 3 DI water. After reaction, the acid orange 7 dye was desorbed by placing the NPs in pH 12 DI water for 30 min. The absorbance was measured at a wavelength of 485 nm using a spectrophotometer (UV-1650PC, Shimadzu, Japan).

### 2.4. Surface Coating of PCL Scaffold with HAp and Simvastatin Loading

SIM was dissolved in 50% ethanol at 5 wt % concentration and then βCD grafted HAp NPs (3 mg/mL) were suspended in the solution. AD-modified PCL scaffolds were immersed in the aforementioned solution and then subjected to ultra-sonication at room temperature for 1 h. SIM was loaded into the βCDs of the HAp NPs that were attached to ADs on the surface of the PCL scaffold. After the reaction, the surface of the PCL scaffolds was washed to remove any left over, unloaded HAp NPs and SIM. After 14 days of incubating SIM-loaded samples in a 15 mL conical tube containing 3 mL PBS (pH 7.4) at 37 °C with orbital agitation at 100 rpm, the solution containing released SIM was freeze-dried and redissolved in 1 mL EtOH. The loading amount of SIM was analyzed by ultraviolet-visible (UV) spectroscopy (UV-1650PC, SHIMADZU, Kyoto, Japan) at a wavelength of 238 nm and compared to a calibration curve of standard solution at a certain concentration of SIM.

### 2.5. Characterization of βCD-Grafted HAp and PCL Scaffolds

The surface modified HAp NPs were characterized by thermal gravimetric analysis (TGA, TGA Q5000 IR/SDT Q600, TA Instruments, New Castle, DE, USA) in a scanning range from 25–800 °C at a constant heating rate of 5 °C min^−1^. The surface morphology of HAp-coated PCL scaffolds was analyzed with a scanning electron microscope (SEM, Hitachi S-2300, Tokyo, Japan). Images of samples were taken with 15 kV of an accelerating voltage after sputter-coating with gold. Surface chemical compositions of βCD-modified HAp and AAc-grafted PCL scaffolds (diameter: 8 mm and thickness: 3 mm) were determined using an X-ray photoelectron spectroscope (XPS, Thermo Fisher Scientific, MA, USA) at a grazing angle of 90° under high vacuum (<3.1 × 10^−9^ Torr.). Monochromatic aluminum *K*α X-ray radiation (photoelectron energy = 1486.6 eV) was used and the wide-scanned XPS spectra were obtained at a pass energy of 187.8 eV.

### 2.6. Cell Culture

Human adipose-derived stem cells (hADSCs) were cultured in MesenPRO RSTM medium (MPRO medium, Invitrogen, Carlsbad, CA, USA). To test cell proliferation, hADSCs were seeded on sample PCL scaffolds (*n* = 4) with density of 1 × 10^5^ cells/scaffold and cultured in 48-well plates in a humidified incubator (Thermo Fisher Scientific, MA, USA) at 37 °C and 5% CO_2_ for 1, 4, and 7 days. To examine osteogenic differentiation, hADSCs were seeded onto PCL scaffolds with a density of 1 × 10^5^ cells/scaffold. 1 day after seeding, hADSC-seeded PCL scaffolds were cultured with osteogenic differentiation medium (MesenPRO RSTM medium supplemented with 10 nM dexamethasone, 25 µg/mL l-ascorbic acid, and 10 mM β-glycerophosphate) for 7, 14, and 21 days. The levels of alkaline phosphatase (ALP) in the differentiated hADSCs cultured on PCL scaffolds were measured using spectroscopy. Briefly, aliquots (50 μL) of each sample were incubated in 5 mM *p*-nitrophenol phosphate solution (Sigma, St. Louis, MO, USA) for 30 min at 37 °C. The level of *p*-nitrophenol production due to the presence of ALP was measured at 410 nm using a microplate reader (Bio-Rad, Hercules, CA, USA).

### 2.7. In Vivo Animal Study

Male New Zealand rabbits (approximately 2.5 kg) were used as an *in vivo* model to examine bone tissue regeneration. Animal selection, management, and surgery protocol were approved by the Kyung Hee Medical Center Institutional Animal Care and Use Committee, Seoul, Korea (KHMC_IACUC 11-014). Four circular bone defects of 8 mm in diameter were generated in the cortical bone of a rabbit by pushing a trephine bar through both sides of the inter-parietal suture lines of the cranium without harming the meningeal membrane. PCL scaffolds (8 mm in a diameter, 3 mm in a thickness, *n* = 3 per group) were implanted into the defected sites. Each rabbit was anesthetized, sacrificed, and the parietal bone was harvested 6 weeks post implantation. Micro computed tomography (μCT) (a SkyScan1173, SKYSCAN, Kartuizersweg 3B 2550, Kontich, Belgium) was used with settings of 130 kV, 30 μA current, and 250 ms of exposure time to evaluate regeneration of defected bones. The scanned images were reconstructed using NRECON (version 1.6.3.2, Skyscan, Kontich, Belgium, pixel size = 13.85 µm, angular Step = 0.2°, frame averaging = 5, beam hardening correction = 40%) and 3D images were analyzed using CTAn software (CT Analyser, version 1.14.4.1, Skyscan, Kontich, Belgium), to quantify the total volume of newly formed bone as mean gray value and standard deviation of the region of interest (ROI) from multiplanar reconstructed images.

### 2.8. Statistical Analysis

Statistical analysis was performed using PASW Statistics 18 software (SPSS, Inc., Chicago, IL, USA). All values are expressed as means ± standard deviations and differences, and *p*-values less than 0.05 were considered statistically significant.

## 3. Results and Discussion

### 3.1. Characterization of PCL Scaffolds

**Figure 1:**
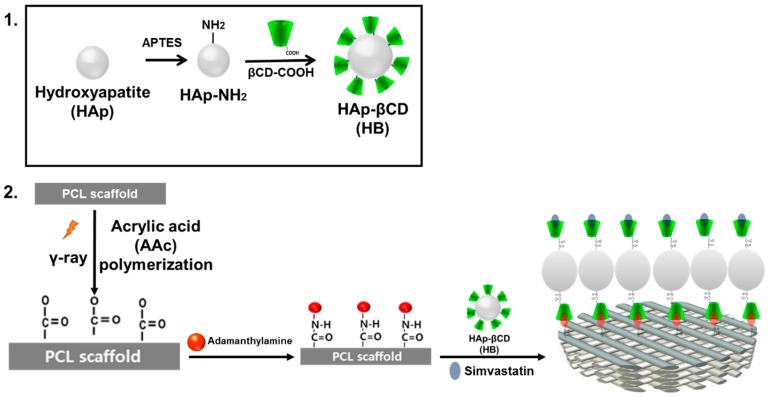
Schematic illustration of hydroxyapatite coated 3D poly(ɛ-caprolactone) (PCL) scaffolds. (**1**) Preparation of β-cyclodextrin (βCD)-grafted hydroxyapatite (HAp) and (**2**) HAp coating and simvastatin loading on the surface of adamantane (AD)-modified PCL scaffold.

**Figure 2:**
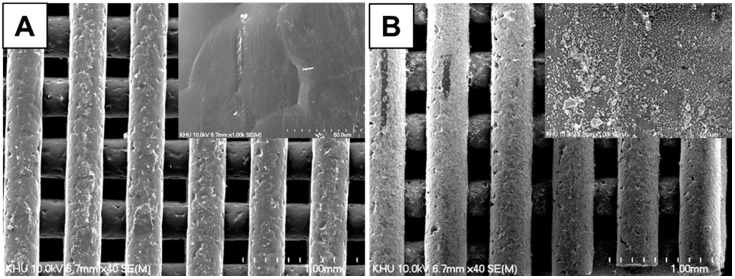
Scanning electron microscopy (SEM) images of β-cyclodextrin (βCD)-grafted hydroxyapatite (HAp)-coated poly(ɛ-caprolactone) (PCL) scaffolds (**A**) without and (**B**) with adamantane (AD) grafting.

Tremendous efforts have been made to develop ECM mimetic scaffolds in the setting of regenerative medicine [23]. As part of ongoing efforts, we developed HAp-modified PCL scaffolds to represent structural elements of native bone and loaded an osteoinductive drug using an inclusion complex system. In particular, SIM was loaded to recapture osteoinductive effects of the biochemical factors that instruct ECM dynamics in the bone niche (Figure 1). The SEM images of HAp-coated PCL scaffolds are shown in Figure 2. The 3D scaffold structures had highly regular pores of >600 µm, porosity of 91.15% and strands with a thickness of 380–400 µm [24]. After βCD-modified HAp coating, a homogeneous distribution of HAp NPs along the surface was observed on the AD-modified PCL scaffold surface whereas βCD-modified HAp NPs were not observed on non-coated PCL scaffolds. 

Simulated body fluid (SBF) solution immersion has been commonly used to produce an osteoconductive surface for bone tissue engineering [24,25]. However, its coating procedures, such as using SBF solutions and direct coating of HAp powder, have some disadvantages because SFF requires extended periods of time to successfully work; and the surface structure of the scaffolds can be changed via indiscreet mineral formation, resulting in irreversible alteration of scaffold properties. To overcome these disadvantages, we used βCD-modified HAp as a coating material and applied this strategy to surface coating of PCL scaffold by an inclusion complex system between βCD on the HAp and AD on the PCL scaffold. This reaction produced a homogeneous coating of HAp NPs on the PCL scaffold and enhanced the loading efficiency of the drug [26].

### 3.2. Characterization of βCD-Modified HAp and AD-Modified PCL Scaffold

**Figure 3:**
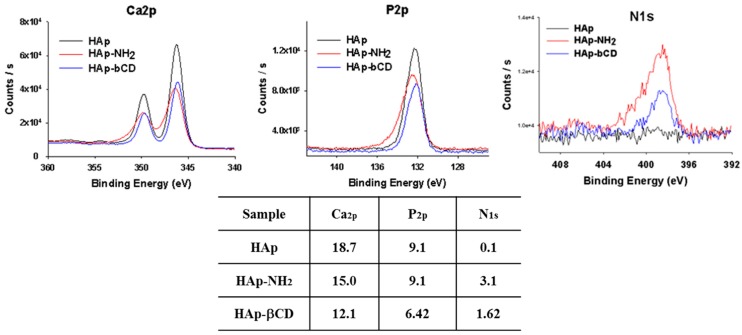
X-ray photoelectron spectroscopy (XPS) analysis of β-cyclodextrin (βCD) grafted Hydroxyapatite (HAp), 3-aminopropyltriethoxysilane (APTES) grafted HAp, and pure HAp powders.

**Figure 4:**
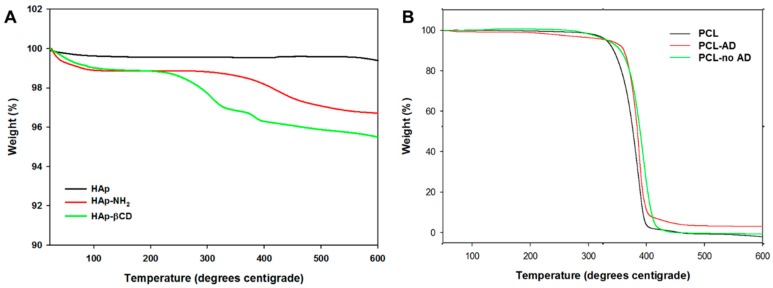
Thermal gravimetric analysis (TGA) of (**A**) surface-modified hydroxyapatite (HAp) composites and (**B**) HAp-coated poly(ɛ-caprolactone) (PCL) scaffolds.

To analyze the surface characteristics of βCD-modified HAp and AD-modified PCL scaffolds, X-ray photoelectron spectroscopy (XPS) was conducted on these samples. After amino-functionalization of HAp and βCD, the binding energy peaks of Ca2p, P2p, and N1s of HAp NPs were changed (Figure 3) as evidenced by the facts that the Ca2p and P2p peaks of pure HAp were reduced; and the N1s peak was changed from 0.1 eV of HAp to 3.1 eV and 1.6 eV for HAp-NH_2_, and HAp-βCD, respectively. These results confirmed that the HAp surface was successfully amino-functionalized by APTES, leaving NH_2_ groups for grafting of βCD onto the HAp NPs. Table 1 shows the quantified amine concentrations on the amino-functionalization of HAp. The amine concentration of HAp-βCD was 3.5 ± 0.4 μmol/mg and APTES-grafted HAp was 21.9 ± 2.9 μmol/mg. These results showed approximately 18.4 μmol/mg of NH_2_ groups were generated to graft βCD onto the surface of the HAp NPs. Unexpectedly, some remaining primary amines of the HAp NPs were detected after βCD grafting when fluorescent amine was used (data not shown), indicating the need to improve the βCD grafting efficiency further. To determine the amount of the grafted βCD on the HAp NPs, both non-treated HAp and HAp-βCD were analyzed by thermal gravimetric analysis (TGA) at a temperature range of 0–800 °C (Figure 4A). The TGA curve of both non-treated HAp and HAp-βCD showed weight loss at a temperature range of 20–100 °C as expected given that the scaffolds have some moisture content that can evaporate within this temperature range. Between 300 and 600 °C, weight loss of HAp-βCD was observed due to thermal degradation of grafted βCD, confirming the presence of grafted βCD.

**Table 1:** Amine contents of 3-aminopropyltriethoxysilane (APTES)-grafted hydroxyapatite (HAp) and β-cyclodextrin (βCD)-grafted HAp.

Heading	Hap-NH_2_	Hap-βCD
Amine contents (μmol/mg)	21.9 ± 2.9	3.5 ± 0.4

To determine the degree of AD grafting on to the PCL scaffold surface, acrylic acid (AAc) was grafted onto the surface as the functionalized carboxyl groups could then be stained by toluidine blue (Figure S1). Using the same carboxyl groups, ADs were grafted onto the surface of the PCL scaffold. Figure S2 shows the amount of grafted AD resulting from various AD concentrations. Nearly 100% of AD in all treatment groups was grafted on the surface of the PCL scaffold regardless of the solution concentration. To measure the amount of coated HAp on the PCL scaffolds, TGA was conducted at a temperature range of 0–600 °C (Figure 4B). HAp was found to be 7 wt % whereas 0.7 wt % was reported for non-AD grafted and 0.2 wt % for the PCL control.

### 3.3. In Vitro Cell Test of PCL Scaffolds

**Figure 5:**
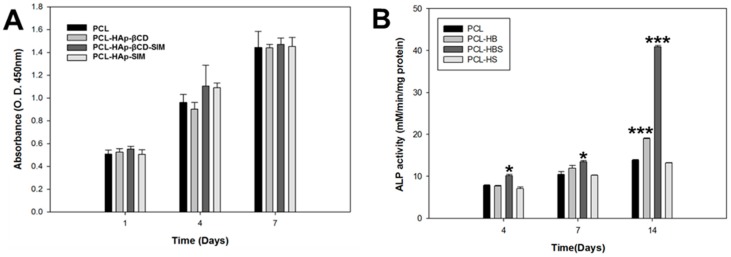
Human adipose-derived stem cell (hADSC) (**A**) proliferation and (**B**) alkaline phosphatase (ALP) activity on poly(ɛ-caprolactone) (PCL) scaffolds.

The initial loading amount of SIM on the HAp-βCD-coated PCL scaffold was 9.56 µg/mg, and 100% of SIM was released after 14 days (data not shown). Proliferation of hADSCs was measured using the CCK-8 assay kit at 1, 4, and 7 days post culture. Figure 5A shows that there was no observed cytotoxicity among all groups and demonstrated similar proliferation patterns. Figure 5B shows ALP activities of hADSCs on the test PCL scaffolds. Commonly, ALP activity is an early marker of immature osteoblast activity. Moreover, it has been described that ALP cleavage of organic phosphate plays a role in the mineralization of the extracellular collagenous matrix by providing calcium and phosphate ions to generate new formation of the cell-mediated calcium phosphate mineralized matrix [25,26]. Elevated levels of ALP activity were noticed on HAp-coated PCL and SIM-loaded HAp-coated PCL scaffolds at day 14. The ALP activity was significantly higher on the HAp-coated PCL and SIM-loaded HAp-coated PCL scaffolds than the PCL control scaffold at 14 days of cell culture.

### 3.4. In vivo Animal Study

**Figure 6:**
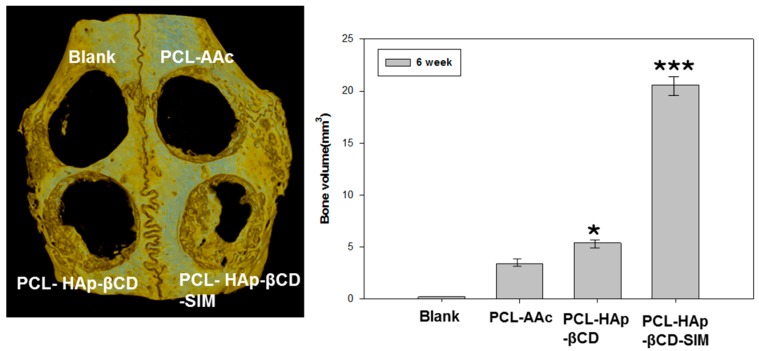
Micro-computerized tomography (μCT) scan images of rabbit calvarial defects after 6 weeks of implantation of various poly(ɛ-caprolactone) (PCL) scaffolds. * *p* < 0.05 and *** *p* < 0.005 compared to the PCL-AAc group.

The bone defect repair effect of scaffolds was investigated using the rabbit calvarial defect model by *in vivo* experiments. Figure 6 illustrates the raw µCT images and the corresponding analysis. HAp-coated and SIM-loaded HAp-coated PCL scaffold groups had noticeable increases in bone formation after 6 weeks of implantation as compared to the PCL control group. Using image analysis software, an 8 mm circular ROI was drawn on the µCT images over the defect site; and the regenerated total bone volume (RBV) was obtained by measuring the mean gray value for this region. The total RBV of all groups had increased over 6 weeks, however the RBV in the SIM-HAp loaded PCL was 22.3 ± 1.9 mm^3^ at 6 weeks, which was significantly greater than that observed in the PCL scaffolds groups. Bone regeneration in the defects was found in both HAp-coated and SIM-loaded HAp-coated PCL scaffolds. However the SIM-loaded HAp-coated PCL scaffold group demonstrated more active osteogenesis in the defect area compared with other groups during inplantation periods. The incorporation of SIM and HAp was shown to significantly increase the bone regeneration *in vivo*. HAp NPs provide a suitable microenvironment for mimicking the inorganic phase of the native tissue and SIM has a positive influence on accelerating mineral deposition *in vivo*. SIM is widely used in clinical lipid-lowering drugs, and it plays a role in promoting new bone formation and regulation by BMP-2 and VEGF expression of osteoblasts [27,28].

## 4. Conclusions

In this study, we developed PCL scaffolds using a 3D plotting system for bone tissue engineering. We coated PCL scaffolds with βCD-grafted Hap NPs, followed by SIM loading, using an inclusion complex system. The results indicate that βCD grafting enhanced HAp coating to the PCL surface as well as the SIM loading efficiency compared to non-grafted PCL scaffolds. The SIM-loaded PCL scaffold also enhanced the growth and osteogenic differentiation of hADSCs *in vitro* and bone regeneration *in vivo*, suggesting the SIM/HAp loaded PCL scaffold as a promising construct for bone tissue engineering.

## Supplementary Materials

The following are available online at www.mdpi.com/2073-4360/8/2/49/s1. Figure S1: Schematic illustration of carboxyl group modified PCL scaffold via gamma ray treatment and representative toluidine blue assay stained PCL scaffold, Figure S2: The amount of grafted ADs on the surface PCL scaffolds by treated various concentration of adamantylamine.
