# Supplementary material for: Development of Poly(ɛ-Caprolactone) Scaffold Loaded with Simvastatin and Beta-Cyclodextrin Modified Hydroxyapatite Inclusion Complex for Bone Tissue Engineering
Source: Polymers (Basel). 2016 Feb 9;8(2):49. doi: 10.3390/polym8020049 (PMC6432564; doi:10.3390/polym8020049)
Supplement: Supplementary file 1 [file polymers-08-00049-s001.pdf]

Supplementary Materials: Development of Poly(ε-Caprolactone) Scaffold Loaded with Simvastatin and Beta-Cyclodextrin Modified Hydroxyapatite Inclusion Complex for Bone Tissue Engineering

Jung Bok Lee, Ji Eun Kim, Min Soo Bae, Su A Park, Daniel A. Balikov, Hak-joon Sung, Hoon Bong Jeon, Hun Kuk Park, Kook Sun Lee, Il Keun Kwon

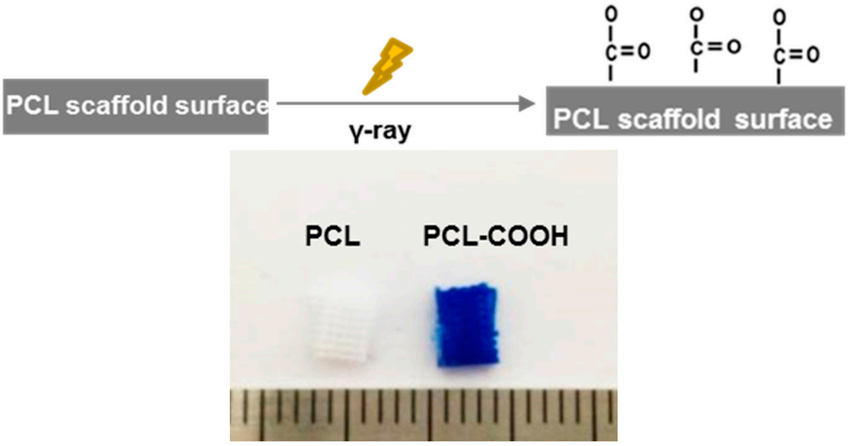

Figure S1. Schematic illustration of carboxyl group modified PCL scaffold via gamma ray treatment and representative toluidine blue assay stained PCL scaffold.

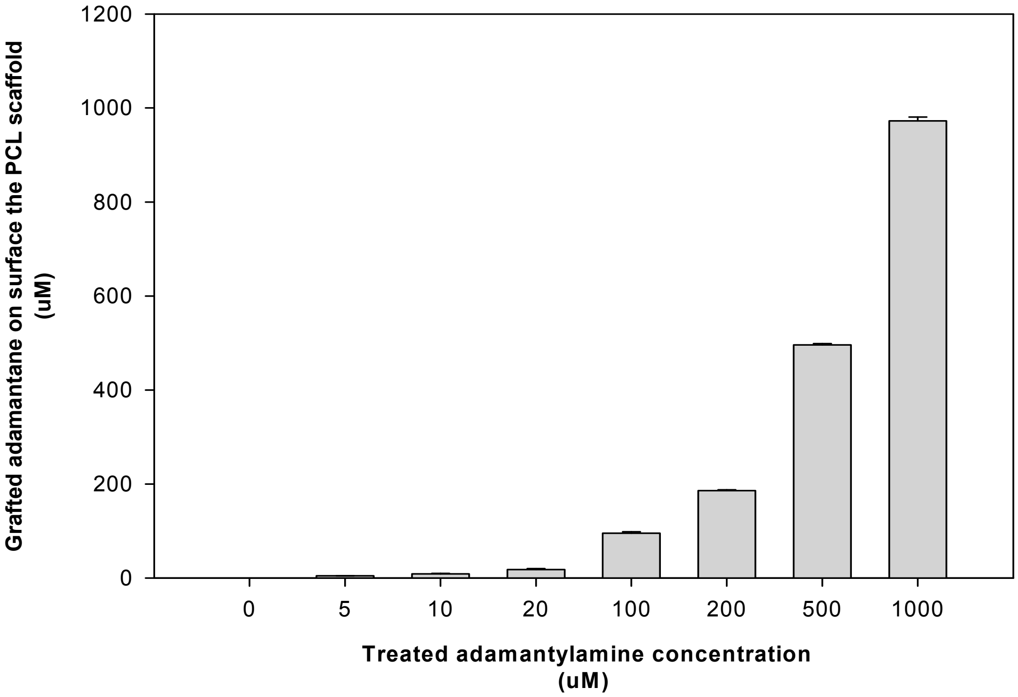

Figure S2. The amount of grafted ADs on the surface PCL scaffolds by treated various concentration of adamantylamine.

© 2016 by the authors; licensee MDPI, Basel, Switzerland. This article is an open access article distributed under the terms and conditions of the Creative Commons by Attribution (CC-BY) license (http://creativecommons.org/licenses/by/4.0/).
